# The Psychological Impact of Hand Injuries Among Foreign Workers in Singapore

**DOI:** 10.7759/cureus.60772

**Published:** 2024-05-21

**Authors:** Ian Dhanaraj, Vaikunthan Rajaratnam, Hasif Jaafar, Karen Morgan

**Affiliations:** 1 Orthopaedic Surgery, Woodlands Health, Singapore, SGP; 2 Hand Surgery Unit-Orthopaedic Surgery Department, Khoo Teck Phuat Hospital, Yishun, SGP; 3 Academy of Contemporary Islamic Studies, Universiti Teknologi MARA, Negeri Sembilan, MYS; 4 Psychology and Behavioural Science, The Royal College of Surgeons in Ireland and University College Dublin (RCSI & UCD) Malaysia Campus, Penang, MYS

**Keywords:** psychosocial, foreign worker, stress, anxiety, depression, migrant workforce, hand injury

## Abstract

Introduction: Foreign workers comprise a significant portion of Singapore’s workforce. They face multiple challenges when working there. A hand injury may add to these stressors, causing profound psychological and social impact. There are few studies in the literature that have analyzed this effect. The primary objective of this study, the first of its kind in Singapore, is to investigate the psychological impact and functional outcomes of hand injuries among foreign workers. By assessing the prevalence of psychological conditions such as stress, anxiety, and depression, along with measuring functional impairment using validated tools, this study aims to provide insights into the psycho-social challenges faced by this vulnerable population.

Methods: A single-encounter interview was conducted for eligible patients. Psychological impact was measured using the DASS-21, symptom severity and function with the QuickDASH, and pain with the VAS. Injury-specific and demographic data were also collected. The Mann-Whitney U test and the Chi-Squared test were applied for non-parametric variables and categorical data, respectively. The adjusted p-value was <0.05.

Results: Eighty foreign workers were recruited. The mean age was 33 years, and the median age was 31.5 years (28.2 to 37.0). The majority were male (97.5%), married (60%), and had a salary of less than SGD1500 (USD1077) per month (81.3%). The most common mechanism of injury was penetrating (60%, n=48). Stress, anxiety, and depression were positively associated with limitations in daily function. Multivariate analysis found that limitations in daily function were independently associated with stress, anxiety, and depression, regardless of hand dominance.

Conclusion: This study has shown a significant psychological and social impact of hand injuries among foreign workers in Singapore. There is potential for the development of screening and support programs for at-risk workers to cater to their mental well-being. We recommend that the psychological impact of hand injuries be factored into holistic management and rehabilitation with adequate time and resource allocation. An ancillary benefit is the improvement of productivity and overall contribution to Singapore’s economy.

## Introduction

Foreign workers are essential to Singapore’s workforce. As of June 2019, there were 981,000 foreign workers in Singapore, making up 26.8% of the total workforce [1].

Foreign workers have vocations ranging from construction and shipyard work to domestic duties within households. Many are unskilled, have low levels of education, and come from low socio-economic backgrounds in developing countries. They are uprooted from their families and communities and have the added burden of language and cultural barriers. They may also experience financial constraints, long working hours, and occasional mistreatment. To gain employment in Singapore, foreign workers require work permits [1]. This is the bottom-tier employment visa in Singapore assigned to low-skilled workers [2]. The visa of a work permit holder and their employment contract are closely linked. Workers are, therefore, heavily dependent on their employers, who hold influence over possible repatriation [2]. Given that their vocations predominantly involve manual labor, sustaining hand injuries in the line of duty is not uncommon for foreign workers [1]. Studies have shown that a significant proportion of acute hand trauma results from occupational injuries among manual workers [3]. In addition to the above-mentioned stressors, an injured hand may result in significant negative psychological effects on the worker.

A person’s hands are their primary means of interacting with their surroundings. An injured or disfigured hand may invite unwanted stares or comments, and this experience can be traumatic. Hand injuries may result in functional, occupational, or social deficits [4]. They may also lead to insecurity and dependency, which may aggravate one’s psychological state. While the primary aim in the management of traumatic hand injuries is musculoskeletal and involves bony and/or soft tissue stabilization or reconstruction, the psychological impact of these injuries must not be neglected.

Psychological trauma following hand injuries is well-established in the literature [5]. Common sequelae include depression, anxiety, and posttraumatic stress disorder (PTSD) [5]. The prevalence of psychological impact is variable due to the different psychological assessment tools used, the heterogeneity of study methodology, and assessments based on subjective patient reporting. The incidence of psychological distress may be as high as 35%, occurring 1-2 weeks after the trauma [6], with functional outcomes correlating closely with psychological impact at the early and late stages of recovery [7].

A local study [8] showed that most patients who attended the emergency department for hand trauma were male, 20-30 years old, and had industrial accidents. Psychological evaluations were not performed. 

Most studies involving hand injuries among foreign workers focus on the bio-physical aspect of the injuries, medical or surgical management, rehabilitation, and economic or compensation-related issues. Few studies investigate the psychosocial effects of these injuries.

Pain

Generally, pain decreases in the first three months after an injury [4]. Greater and more persistent pain is associated with dominant hand injuries and amputations as compared to other injury types [4]. Himmelstein et al. showed that work-disabled patients had significantly higher pain levels, pain catastrophizing, and fear of pain and reinjury as compared to those who were capable of working [9].

Psychological symptoms

Gustaffson et al. found that patients suffered less anxiety and depression three months following occupational hand injuries as compared to weeks after. Psychological symptoms were more pronounced in those who sustained amputations [4]. A negative effect may indicate general emotional distress and is an important predictor of the delayed and overall outcome of the return to work [10]. Psychological sequelae are present in most patients with work-related hand injuries, regardless of the type of litigation or claim [11].

Function and disability

With regards to psychological sequelae from hand trauma, prompt intervention, symptom management, and systematic rehabilitation allow for an earlier return to work [11]. Multidisciplinary approaches to treat and rehabilitate patients help to decrease anxiety and promote good communication, with an emphasis on patient education [12]. Patients with amputations spend more time away from work, with almost half reporting a worse situation in work, leisure, and overall life upon returning [4]. Injury severity has a significant impact on functional outcome and recovery duration before returning to work. Workers with severe injuries are more than four times more likely to have a delayed recovery [10]. Roesler et al. also established that workers who live alone may worsen their injury by performing tasks beyond their level of recovery, resulting in additional psychological stress [10]. Workers who faulted their company or equipment for their injuries are at a greater risk of not returning to pre-morbid employment [13]. Grunert et al. showed that psychological symptoms do not correlate with the resolution or size of litigation settlements or compensation. In their study, 69% of workers' compensation litigants and 90% of third-party litigants persisted in being psychologically symptomatic six months after litigation was resolved [11].

In Singapore, a unique partnership exists between unions, employers, and the government to enhance Singapore’s economy and foster amicable labor-management relations. One key focus of this tripartite relationship is ensuring fair and progressive employment practices [1]. 

Following a hand injury that impairs the worker’s ability to perform, their work permits may be canceled or unrenewed and converted to a Special Pass visa, which allows them to remain, but not work, in Singapore [1]. During this time, medical leave issued by doctors is essential for foreign workers to receive a salary, which is a contentious issue for both employers and medical professionals. After treatment, workers are assessed for workmen’s compensation, which follows structured guidelines set by the Ministry of Manpower. This assessment may occur months after the initial injury and focuses on biophysical deficits resulting from musculoskeletal injuries, such as numbness or joint stiffness. Psychological illness is not a consideration in the workmen's compensation.

The primary objective of this study, the first of its kind in Singapore, is to investigate the psychological impact and functional outcomes of hand injuries among foreign workers. By assessing the prevalence of psychological conditions such as stress, anxiety, and depression, along with measuring functional impairment using validated tools, this study aims to provide insights into the psycho-social challenges faced by this vulnerable population.

## Materials and methods

This cross-sectional study was approved by the National Healthcare Group Domain Specific Review Board (Ref: 2018/00362), and written informed consent was obtained from all participants.

Consenting foreign workers with Work Permits or Special Passes, aged 21 years and older, with a history of an occupational hand injury (occurring at least one month before consultation), were consecutively sampled and recruited from the hand surgery specialist outpatient clinic between June 2018 and June 2019. They were excluded if they had a pre-existing congenital hand deformity, a prior hand injury or surgery, a self-inflicted injury, associated polytrauma, or a history of psychiatric illness.

A single, in-person interview was conducted by one of the authors. This was done primarily in English, and assistance was sought from hospital-employed, accredited translators as required. Translated versions of the consent and questionnaire forms were also made available. Standard scripts were used for consent, the collection of demographic data, and the administration of the questionnaires. Deidentified forms were manually filled in by participants. The responses were subsequently collected and tabulated.

Information on nationality, age, gender, marital status, family setup, handedness, lodging arrangement, and average monthly salary was collected at enrolment. Other collected data included injury-specific parameters, comprising the site of injury, mechanism of injury, duration since the accident, total days of medical leave or light duties given, and whether any surgery was performed.

All participants were then assessed for psychological impact by using the Depression Anxiety Stress Scale (DASS-21), symptom severity and limitation of daily function by using the Quick Disabilities of the Arm, Shoulder, and Hand (QuickDASH) score, and pain experienced by using the Visual Analogue Scale (VAS).

DASS-21 score

The DASS-21 is a self-reported scale devised to evaluate the perceived severity of symptoms related to the negative emotional states of depression, anxiety, and stress [14]. It allocates seven questionnaire items for each of these emotional states and grades the severity on a five-point scale, ranging from normal to extremely severe [14]. The DASS-21 has been shown in the literature to be a validated and reliable tool in assessing and determining the severity of symptoms related to depression, anxiety, and stress [15]. Participants with severe and extremely severe stress, anxiety, and depression were referred for psychological follow-up.

QuickDASH score

The QuickDASH score was used as a surrogate marker of function in the patient's injured limb. The 11-item self-reported score evaluates various facets of hand disability and function, assesses the patient’s ability to perform dextrous tasks, and interferes with social and occupational activities. QuickDASH is a reliable and valid tool. It has a Cronbach’s alpha of 0.94 and an intraclass correlation coefficient (ICC) of 0.94. Pearson product-moment correlations were derived from tests for convergent validity, which studied VAS for overall problem (r=0.70), overall pain (r=0.73), ability to function (r=0.80), and ability to work (0.76) [16].

VAS

The patient’s pain score specific to the relevant injury at the time of the interview was measured on a 10-point VAS. This tool, which is reliable and widely used in clinical research involving musculoskeletal conditions, is scored based on 10 being the worst possible pain and 0 being no pain.

Data analysis

All statistical analyses were conducted using IBM Corp. Released 2015. IBM SPSS Statistics for Windows, Version 23.0. Armonk, NY: IBM Corp. Categorical variables were presented as frequency and percentages. Continuous variables were reported as a mean and standard deviation. A p-value of <0.05 was used to define statistical significance. Participants were grouped according to whether they had injured their dominant or non-dominant hand. Age, QuickDASH, and VAS were non-parametric and therefore expressed as medians with interquartile ranges (IQR) and compared using the Mann-Whitney U test. Other categorical data were analyzed using the chi-square test. The groups differed significantly in age and lodging. The association between DASS-21 and QuickDASH was analyzed using the correlation test and further adjusted for potential confounders using linear regression.

## Results

A total of 80 patients who met the inclusion and exclusion criteria were enrolled in the study. Most patients were male (97.5%, n=78) and were of Indian (35.0%, n=28) and Bangladeshi (33.8%, n=27) nationalities. The majority were married (60.0%, n=48) and were childless (51.0%, n=41).

Most patients were interviewed between four and eight weeks after their injury (75.0%, n=60), and underwent surgical procedures as treatment (95.0%, n=76).

46.3% (n=37) of patients sustained injuries on their dominant hand, with the main mechanisms of injury being penetrating (60.0%, n=48) and crushing (30.0%, n=24). Patients had an average of 41.6 days (0-259) of medical leave and 19.5 days (0-143) of light duties. The main outcome measure of this study was the psychological impact calculated by the DASS-21. Patients who scored normal were considered to have no psychological impact, while those who had symptoms were considered to have a psychological impact. The incidence of the conditions is as follows: depression (45.0%, n=36), anxiety (51.3%, n=41), and stress (48.8%, n=39).

Table 1 highlights the characteristics of participants with dominant and non-dominant hand injuries. 

**Table 1:** Results: Demographics, DASS-21, QuickDASH, and VAS Comparison of the characteristics of those who had dominant and non-dominant hand injuries.

Variable**	Dominant Hand Injury, n = 37 (46.3%)	Non-Dominant Hand Injury, n = 43 (53.8%)	p-value
Age, median (IQR)***	30 (26.0 – 35.5)	34 (29.0 – 42.0)	0.004*
Gender			
Male	36 (97.3)	42 (97.7)	0.914
Female	1 (2.7)	1 (2.3)	
Nationality			
Indian	15 (40.5)	13 (30.2)	0.294
Bangladeshi	15 (40.5)	12 (27.9)	
Malaysian	3 (8.1)	8 (18.6)	
Chinese	3 (8.1)	8 (18.6)	
Others	1 (2.7)	2 (4.7)	
Marital status			
Married	19 (51.4)	29 (67.4)	0.177
Single	18 (48.6)	13 (30.2)	
Divorced	0 (0)	1 (2.3)	
Number of Children			
0	23 (62.2)	18 (41.9)	0.170
1	9 (24.3)	12 (27.9)	
2	3 (8.1)	9 (20.9)	
≥3	2 (5.4)	4 (9.3)	
Lodging			
Dorm	33 (89.2)	28 (65.1)	0.026*
Company house	0 (0)	4 (9.3)	
Others	4 (10.8)	11 (25.6)	
Salary per month			
<1000	16 (43.2)	15 (34.9)	0.503
1000 – 1500	16 (43.2)	18 (41.9)	
>1500	5 (13.5)	10 (23.3)	
Injury mechanism			
Penetrating	21 (56.8)	27 (62.8)	0.225
Crushing	10 (27.0)	14 (32.6)	
Others	6 (16.2)	2 (4.7)	
Duration since injury			
<8 weeks	25 (67.6)	27 (62.8)	0.632
8 - 16 weeks	9 (24.3)	14 (32.6)	
>16 weeks	3 (8.1)	2 (4.7)	
Had surgery			
Yes	35 (94.6)	41 (95.3)	0.877
No	2 (5.4)	2 (4.7)	
Translator needed			
Yes	5 (13.5)	6 (14.0)	0.955
No	32 (86.5)	37 (86.0)	
Depression			
Normal	21 (56.8)	23 (53.5)	0.100
Mild	3 (8.1)	10 (23.3)	
Moderate	8 (21.6)	8 (18.6)	
Severe	4 (10.8)	0 (0)	
Extremely severe	1 (2.7)	2 (4.7)	
No depression	21 (56.8)	23 (53.5)	0.770
Depression	16 (43.2)	20 (46.5)	
Anxiety			
Normal	18 (48.6)	21 (48.8)	0.676
Mild	5 (13.5)	8 (18.6)	
Moderate	8 (21.6)	10 (23.3)	
Severe	5 (13.5)	2 (4.7)	
Extremely severe	1 (2.7)	2 (4.7)	
No anxiety	18 (48.6)	21 (48.8)	0.987
Anxiety	19 (51.4)	22 (51.2)	
Stress			
Normal	21 (56.8)	20 (46.5)	0.303
Mild	5 (13.5)	13 (30.2)	
Moderate	6 (16.2)	4 (9.3)	
Severe	5 (13.5)	6 (14.0)	
No stress	21 (56.8)	20 (46.5)	0.361
Stress	16 (43.2)	23 (53.5)	
QuickDASH, median (IQR)***	56.8 (26.1 – 76.1)	59.1 (34.1 – 75.0)	0.589
VAS, median (IQR)***	6.0 (3.0 – 8.0)	5.0 (2.0 – 8.0)	0.133
*Significant at p<0.001, **Chi-Square Test, ***Mann-Whitney U Test			

The median age (IQR) of all participants was 31.5 (28.2 to 37.0) years, with a higher median age among the non-dominant hand injury group compared with the dominant hand injury group (p=0.004). Participants with dominant hand injuries were more likely to stay in a dormitory (p=0.026).

There were no differences between groups regarding gender, nationality, marital status, number of children, monthly salary, injury mechanism, duration since injury, surgery performed, or need for a translator. There were no differences in terms of depression (p=0.770), anxiety (p=0.987), or stress (p=0.361). The median score for the QuickDASH and VAS of all the participants was 56.8 (31.8 to 75.0) and 5.0 (2.0 to 8.0), respectively. No significant differences were found between the groups in QuickDASH scores (p=0.589) or VAS (p=0.133).

The association of DASS-21 and QuickDASH based on the side of the hand injury is summarized in Table 2. 

**Table 2:** Correlations of DASS-21 with QuickDASH among dominant and non-dominant hand injuries

	Dominant Hand Injury				Non-Dominant Hand Injury			
DASS-21	QUICKDASH	p-value	Adjusted p-value**	95% Confidence Interval	QUICKDASH	p-value	Adjusted p-value**	95% Confidence Interval
Depression	0.54	0.001*	0.005*	0.05 – 0.25	0.56	<0.001*	0.002*	0.05 – 0.20
Anxiety	0.57	<0.001*	0.015*	0.02 – 0.20	0.46	0.002*	0.002*	0.04 – 0.17
Stress	0.81	<0.001*	<0.001*	0.22 – 0.37	0.42	0.005*	0.001*	0.07 – 0.26
*Significant at p<0.05; **Adjusted with age and lodging								

The presence of these psychological states was shown to have a statistically significant correlation with the functional state and pain, measured by the QuickDASH and VAS, respectively. This is detailed in Tables 2, 3. After adjustment for confounders, for those who had dominant hand injuries, there were significant positive correlations between QuickDASH and depression (r=0.54, adjusted p=0.005) and QuickDASH and anxiety (r=0.57, adjusted p=0.015). Furthermore, there was a strong correlation between QuickDASH and stress (r=0.81, adjusted p=<0.001). In the non-dominant hand injury group, similar results were seen. The QuickDASH correlated well with the depression score (r=0.56, adjusted p=0.002), anxiety score (r=0.46, adjusted p=0.002), and stress score (r=0.42, adjusted p=0.001).

**Table 3:** Correlations of DASS-21 with VAS among dominant and non-dominant hand injuries

	Dominant Hand Injury					Non-Dominant Hand Injury			
DASS-21	VAS	p-value	Adjusted p-value**	95% Confidence Interval	VAS		p-value	Adjusted p-value**	95% Confidence Interval
Depression	0.45	0.006*	0.050	-0.02 – 2.10	0.59		<0.001*	0.001*	0.46 – 1.60
Anxiety	0.33	0.047*	0.301	-0.46 – 1.44	0.40		0.008*	0.014*	0.14 – 1.21
Stress	0.62	<0.001*	0.001*	0.94 – 3.11	0.24		0.115	0.132	-0.19 – 1.40
*Significant at p<0.05; **Adjusted with age and lodging									

Participants with dominant hand injuries showed that depression and anxiety had good correlations with VAS (r=0.45 and r=0.33, respectively). However, when controlled for confounders, they were no longer significant (depression, adjusted p=0.050; anxiety, adjusted p=0.301). On the other hand, participants with a higher stress score tended to have a higher VAS (r=0.62, adjusted p=0.001). In the non-dominant hand injury group, depression and anxiety correlated well with VAS (r=0.59, adjusted p=0.001 and r=0.40, adjusted p=0.014, respectively) but not stress (r=0.24, adjusted p=0.132).

## Discussion

A Boolean search performed on existing literature on PubMed for the terms “workers” and “hand injuries” produced 306 studies, of which only six measured psychological outcomes, as summarized in Table 4. 

**Table 4:** Boolean search, indicating the six studies that included psychological outcome measures.

Study	Total no of workers	Type of injuries	Psych assessment tool	Prevalence of psych impact
Gustafasson (2004) [4]	91	Traumatic and work related	IES, HADS	7/91 (7.7%)
Roesler (2013) [10]	192	Traumatic and work related	PANAS	9/192 (4.7%) PTSD
Bonzani (1997) [12]	50	Work-related upper limb disorders	Clinical assessment	29/50 (58%) mild 17/50 (34%) mod 4/50 (8%) severe
Himmelsteein (1995) [9]	124	Traumatic and work related	Coping strategies questionnaire	19/124 (15%) psych diagnosis 15/124 (12%) depression
Grunert (1991) [11]	143	Traumatic and work related	DSM III-R	100% PTSD (study effects of litigation)
Rusch (2003) [13]	92	Traumatic and work related	DSM IV	70/92 (76%) PTSD 22/92 (24%) Depression

Following a traumatic hand injury, patients may experience an array of psychological distress. However, some patients may fail to exhibit or experience manifestations of these conditions. Factors that have been purported to influence this include fear of job security, liability for injury, litigation, and compensation claims [17].

This study shows that nearly 50% of patients who sustained work-related hand injuries had some degree of psychological impairment. It is difficult to make a meaningful comparison with other studies due to the heterogeneity of methodologies and the psychological assessment tools used. However, this study has shown a higher incidence of common post-traumatic psychological sequelae than what is reported in the literature. Most patients who were surveyed sustained injuries that required surgical intervention. The process itself may be traumatic and contribute to the worker’s psychological state. Being interviewed by a surgeon in a hospital setting may also contribute to the recollection of their injury and the experience of surgery. Baseline data on the incidence of psychological illness among migrant workers in Singapore is currently not available. Existing baseline figures internationally are variable. A systematic review by Foo et al. [18] estimated the prevalence of depression among migrants in general to be 15.6%; however, this did not specifically include laborers, who are the main demographic of our study.

Acute stress reactions are estimated to have an incidence of between 5 and 20% [19]. Post-traumatic stress and depression may persist in up to 50% and 20% of patients at one year, respectively [7]. The estimated overall incidence of psychological sequelae is about 35% [7].

Considering that patients may either intentionally or unintentionally mask their psychological sequelae, the incidence of post-traumatic psychological conditions, as discussed in this study, may be underestimated.

The study displays that the existence of these psychological states following a traumatic hand injury is significantly correlated with the pain score. This is consistent with the literature, which has shown elevated pain scores or persistent pain to be associated with greater levels of anxiety and depression [20]. The recovery and rehabilitation of patients with traumatic hand injuries are known to be negatively influenced by the presence of psychological insults, with which there is an association with chronic pain and permanent disability [21]. Factors that contribute to worse pain scores include dominant hand injuries and poor social support [20]. Multifaceted approaches to reducing pain symptoms have been documented to substantially enhance the well-being and quality of life of affected individuals [22]. Surgeons are often unable to adequately address or treat pain, which may go unrecognized and become chronic [23]. It is therefore prudent to ensure that pain management is emphasized as a priority in the acute setting. Individuals with intractable pain and those at risk of developing chronic pain should be referred at an early stage to a pain specialist and a psychological medicine professional.

Studies have concluded that the psychological impact after hand injuries corresponds closely with functional outcomes at both acute and later stages of recovery [7]. This relationship is also observed in compressive neuropathies such as carpal tunnel syndrome [24]. This study’s findings are consistent with this, as depression, anxiety, and stress among patients were shown to significantly correlate with QuickDASH scores. Though not utilized in this study, quality of life scores such as the Short Form 36 (SF-36), which assesses multiple domains of general health, are also negatively affected by post-traumatic psychological injury [25].

This study is the first in Singapore to evaluate the psychological impact of hand injuries among foreign workers and is performed at a single center with questionnaires administered by a single author, resulting in less variation in protocols and practices that may present confounders or bias. Validated outcome measures were employed as part of the study.

A limitation of the study is language barriers. Questionnaires were conducted in English for the majority of patients who were fluent. Although accredited translators assisted with interviews for patients who were not fluent in English, certain intricacies in administering the psychological questionnaire may be difficult to convey or be lost in translation. Subjects may also be less forthcoming in the presence of a third party, resulting in response bias. Ideally, the interview should be conducted and administered by someone who speaks the participant's native tongue. The study sample was skewed towards young male laborers, who comprise the majority of the foreign worker demographic in Singapore, thereby limiting the generalizability of these results to the broader population.

The findings from the study have also prompted us to establish practice recommendations to better address the mental well-being of such foreign workers. These include 1) a shift in clinical practice among hand surgeons and therapists to accommodate psychological screenings for foreign workers with hand injuries. 2) Collaboration with psychologists and psychiatrists to improve on and develop screening tools, referral workflows, or clinical pathways to allow for a more integrated management of affected workers. 3) Psychological impairment should be factored into the holistic management and rehabilitation of foreign workers with adequate time and resource allocation. 4) The development of psychological support programs for at-risk workers to cater to their mental well-being a basic human right. 5) Psychological impairment should be included in workplace injury compensation assessments, which currently only consider musculoskeletal injuries and disability. 6) Public health policy revisions to allow for psychological illness coverage in healthcare plans purchased for these workers.

## Conclusions

Psychological impairment is common after traumatic hand injuries. Various predisposing and precipitating factors, including patient characteristics, the nature of the injury, and socio-economic factors, may contribute to this psychological impairment. Our study estimates that nearly half of the patients experience psychological impacts such as depression, anxiety, stress, or a combination thereof. It is essential to place emphasis on and cater to the mental well-being of foreign workers. An ancillary benefit of prioritizing the mental well-being of foreign workers is the improvement of productivity and their overall contribution to Singapore’s economy. Therefore, this data may prove useful to employers and government agencies, such as the Ministry of Manpower, for informed decision-making.
